# Dermatology Appointments as an Indicator of Systemic Healthcare Issues

**DOI:** 10.7759/cureus.101556

**Published:** 2026-01-14

**Authors:** Lillian V Rivera, Reina M Gonzalez, Lorna M Torres, Maria Vazquez-Machado, Chavely Calderon, Maria Limardo, Myrta Rivera, Jashira Babilonia, Andrea Becemberg, Nestor Sánchez Colón

**Affiliations:** 1 Dermatology, Ponce Health Sciences University, Ponce, PRI

**Keywords:** dermatology life quality index (dlqi), dermatology workforce shortage, patient quality of life, underserved populations, wait times/access to care

## Abstract

Introduction

Puerto Rico (PR), a territory of the US, faces economic challenges that have resulted in healthcare strain and a shortage of dermatologists. This shortage, coupled with a growing aging population, has led to prolonged wait times for dermatology appointments. These delays negatively affect patients’ quality of life (QOL), especially in underserved areas.

Methods

This cross-sectional study assessed wait times for dermatology appointments in PR and their impact on patient QOL. During the summer of 2023, 464 participants from 12 dermatology clinics completed a questionnaire that included the Dermatology Life Quality Index (DLQI). The survey collected data on demographics, wait times, and the use of over-the-counter remedies. Statistical analyses, including t-tests and chi-squared tests, were used to examine differences between groups based on gender and type of service.

Results

The study revealed that 55% of participants (n = 255) waited more than three months for an appointment, with an average wait time of 2.5 months (SD = 2.49). Women had longer wait times than men (2.59 vs. 2.30 months, p < 0.05). More than 38.7% of patients (n = 179) reported worsening symptoms during the waiting period, and 65.5% (n = 305) resorted to over-the-counter medications or herbal remedies. DLQI scores indicated that 40% of participants (n = 186) experienced moderate to severe effects on their QOL, with women (mean DLQI score = 6.24, SD = 6.46) reporting a greater impact than men (mean DLQI score = 4.80, SD = 5.59; p = 0.008). Patients seeking medical dermatology services had significantly higher DLQI scores (6.24 vs. 3.38; p < 0.001), indicating a greater impact on QOL.

Conclusions

This study highlights the detrimental effects of prolonged wait times for dermatology appointments. The findings emphasize the need to address the dermatology workforce shortage, which disproportionately impacts rural and underserved populations. Strategic efforts to increase access to dermatology care are essential for improving patient outcomes and reducing healthcare disparities.

## Introduction

Puerto Rico’s (PR) prolonged economic recession has caused a significant loss of healthcare personnel, including physicians and nurses, raising serious concerns among government entities and patients [1-3]. Patients report that the decline in healthcare providers has resulted in longer appointment wait times and diminished access to medical care. According to the American Association of Medical Colleges Puerto Rico Physician Workforce Profile, there are 74 active dermatologists in PR, including seven Mohs surgeons, five dermatopathologists, and two pediatric dermatologists [4]. The number of dermatology providers is likely close to or below 1.85 dermatologists per 100,000 inhabitants. The scarcity of dermatologists in this region is frequently raised as a concern during clinical practice. The heightened demand for dermatology services is fueled by an aging population, whose median age has increased by eight years over the past decade, with 28.5% of the population now aged 60 years or older, as well as by an increased incidence of skin cancer and a corresponding rise in service demand [5,6].

Medicaid patients experience longer wait times for healthcare appointments than patients with private insurance [7]. A study in Michigan highlights this problem, showing that Medicaid patients had a mean wait time of 39 days for dermatology appointments, compared with 28 days for those with private insurance [8]. PR’s low reimbursement rates and disparities in Medicaid and Medicare funding allocations represent a significant burden on the healthcare system, contributing substantially to barriers to accessing care [9]. Almost two-thirds of PR’s residents depend on Medicare or local government-sponsored health insurance, including Medicaid.

This issue is not unique to PR but reflects a broader concern recognized nationally in the US. Although the US has experienced an 8% increase in the dermatology workforce, the density of dermatologists still falls short of the recommended 3.77 per 100,000 people as of 2019 [7,8,10,11]. This shortage is evident in the rising national average wait time for dermatology appointments, reported as 34.5 days (1.13 months) in the Merritt Hawkins 2022 Survey and the 2018 Greater Access for Patients Partnership report [12,13]. The problem is further exacerbated by a limited number of dermatology training positions and the accelerated retirement of practicing dermatologists. By 2018, a substantial proportion of dermatologists in the United States were aged 60 years or older, raising concerns about accelerated workforce attrition [4,7]. The growing demand for cosmetic dermatology services also contributes to this issue by reducing the availability of appointments for medical dermatology, thereby further extending wait times [13,14]. Patients with private insurance and those scheduling cosmetic dermatology appointments consistently experience shorter wait times [12,14].

Disparities in wait times can lead to adverse health outcomes, delays in diagnosis and treatment, and increased morbidity and mortality, particularly in conditions such as melanoma, skin cancer, and chronic diseases such as psoriasis. For example, delayed treatment of melanoma is associated with poorer health outcomes and higher mortality rates [13,14]. Similarly, untreated psoriasis is linked to severe comorbidities, including cardiovascular disease, metabolic syndrome, mental health disorders, psoriatic arthritis, certain cancers, and reduced quality of life (QOL) [9,15-17].

Healthcare access encompasses more than hospital availability, physician sufficiency, or insurance status. It refers to the timely use of personal health services to achieve the best possible health outcomes [1]. Investigating wait times in dermatology offers a critical perspective through which the overall effectiveness and efficiency of the healthcare system can be assessed. Prolonged delays in dermatology appointments may reflect systemic issues such as inadequate healthcare infrastructure, workforce shortages, and unequal access to care [18]. These delays can contribute to worsening skin conditions, increased morbidity, and higher healthcare costs. By examining dermatology wait times, policymakers and healthcare providers can identify areas requiring improvement to ensure equitable access to care, enhance patient satisfaction, and support public health goals.

This study was initiated in response to patient concerns regarding access to dermatological care in PR. The study aimed to assess wait times for dermatology appointments in PR, socioeconomic factors contributing to disparities in access, and their impact on participants’ QOL and mental health. We hypothesized that longer wait times for dermatology appointments in PR are associated with a significantly greater impact on patients’ QOL, particularly in relation to stress, emotional health, and symptom severity. The objective of this study was to quantify dermatology appointment wait times in PR and to evaluate their association with patient QOL, perceived stress, emotional health, and self-treatment behaviors.

## Materials and methods

Study design and ethical approval

The study protocol was approved by the Ponce Research Institute Institutional Review Board (approval 2305151721). This exploratory cross-sectional study quantified the average waiting time for dermatology appointments in PR and examined factors influencing access and patients’ perceptions of how waiting affects multiple QOL domains. Specifically, we analyzed whether the association between waiting time and QOL was modified by the type of dermatology service (medical vs. cosmetic) and patient demographics (sex, age, and overall health status).

Survey instrument

A comprehensive, anonymous 35-item questionnaire, available in English and Spanish, was developed to meet the study objectives. It collected information on demographic characteristics, health status, current medications, health insurance, estimated and perceived waiting time for appointments, use of over-the-counter or complementary therapies during the waiting period, and self-reported impacts on symptoms, stress, and QOL.

Dermatology Life Quality Index (DLQI)

Cardiff University, the copyright holder, granted the investigators a free license to use the DLQI (License ID CUQoL3015, issued May 15, 2023). The validated Hispanic Spanish version provided by Cardiff University was used. The DLQI consists of 10 items scored as follows: 0 = “not at all,” 1 = “a little,” 2 = “a lot,” and 3 = “very much.” Item scores are summed to generate a total score ranging from 0 to 30. If exactly one item was missing, the total score was prorated by multiplying the observed score by 10/9; surveys with more than one missing item were excluded.

Total scores were interpreted according to Finlay and Khan: 0-1 = no effect, 2-5 = small effect, 6-10 = moderate effect, 11-20 = very large effect, and 21-30 = extremely large effect on QOL. The DLQI is the most widely used patient-reported outcome measure in randomized controlled trials in dermatology and is available in multiple languages [18]. DLQI scoring followed published guidelines; surveys with more than one missing DLQI item were excluded from analysis, and prorating was applied only when a single item was missing.

Questionnaire development and participant recruitment

The remaining sections of the questionnaire were developed de novo and underwent content validation through patient focus group feedback at the PHSU Dermatology Clinic. The survey was administered in both printed and digital formats. Twelve dermatology clinics across the island participated in recruitment during the summer of 2023. Adults aged 18 years or older who were permanent residents of PR and fluent in Spanish or English were approached in clinic waiting areas and invited to participate.

Statistical analysis

Data were analyzed using MINITAB Version 21 (Minitab, LLC, State College, PA, USA), IBM SPSS Statistics for Windows, Version 29.0 (Released 2022; IBM Corp., Armonk, NY, USA), and Microsoft Excel (Microsoft Corporation, Redmond, WA, USA). Descriptive statistics were used to summarize participant characteristics. Independent-samples t-tests compared mean DLQI scores between men and women and between patients seeking medical versus cosmetic dermatology services. Chi-square tests of independence assessed associations among categorical variables (e.g., service type and sex) and outcomes such as perceived stress, emotional health impact, and DLQI severity categories.

Power analysis

Although no a priori sample size calculation was performed due to the exploratory nature of this cross-sectional study, a post hoc power analysis was conducted. Based on the observed differences in DLQI scores between comparison groups (women vs. men: Cohen’s d = 0.23; medical vs. cosmetic dermatology services: Cohen’s d = 0.46), the final sample size of 464 participants provided more than 80% power to detect small-to-moderate effect sizes at a two-sided α level of 0.05.

## Results

A total of 464 patients were recruited for the study and completed the survey. Of these, 70% were female (Table 1). Regarding geographic distribution, 14% of participants resided in metropolitan areas, while 86% were from nonmetropolitan areas. With respect to visit type, 54% of participants were attending their initial dermatology appointment.

**Table 1:** Demographic data PR, Puerto Rico

Characteristics	n/N (%)
Age, y, mean (SD)	47.3 (20.4)
Sex	N = 464
Female	326 (70.2)
Male	138 (29.7)
Visit type	N = 463
First visit	215 (46.4)
Follow-up	248 (53.6)
Insurance coverage	N = 460
Uninsured	9 (1.9)
Medicare	34 (7.4)
Advantage Medicare	72 (15.7)
PR government insurance (Reforma)	82 (17.8)
Private insurance	263 (57.2)
> 1 health insurance	19 (4.1)
Geographic location	N = 464
Metro area	64 (13.8)
Non-metro area	399 (86.0)
Dermatologic condition	N = 463
First time	265 (57.2)
Preexisting	176 (38.0)
N/A cosmetic service	22 (4.8)
Service needed	N = 463
Medical dermatology	288 (62.2)
Medical + cosmetic dermatology	107 (23.1)
Cosmetic dermatology	68 (14.7)

Most patients (98%) reported having some form of health insurance coverage; of these, 57% had private health insurance and 18% had government-sponsored health insurance. Four percent (4%) reported having more than one health insurance plan, and 2% were uninsured. When asked about the type of service sought, 62% of patients indicated that their visit was exclusively for a medical dermatology complaint, 23% for cosmetic dermatology services, and 15% for both medical and cosmetic dermatology services.

When assessing appointment wait times, 31% of participants reported contacting only one office to secure an appointment, 43% contacted between one and three offices, 16% contacted four to six clinics, 10% contacted more than seven clinics, and 4% contacted more than 10 clinics (Table 2).

**Table 2:** Appointment process data

Variable	Category	n	%
Number of offices called for appointments	1	144	31.2
Number of offices called for appointments	1-3	199	43.1
Number of offices called for appointments	4-9	73	15.8
Number of offices called for appointments	7-9	25	5.4
Number of offices called for appointments	>10	21	4.6
Offices with >6-month wait among those called	To quite a bit (50% of those called)	66	14.2
Offices with >6-month wait among those called	Almost all or most	96	20.7
Offices with >6-month wait among those called	In some (2 or 3 of them)	124	26.7
Offices with >6-month wait among those called	In one only	64	13.8
Offices with >6-month wait among those called	None	104	22.4
Wait time for first appointment (months)	< 1 month	204	44.7
Wait time for first appointment (months)	1-2 months	108	23.7
Wait time for first appointment (months)	2-3 months	1	0.2
Wait time for first appointment (months)	3-4 months	83	18.2
Wait time for first appointment (months)	5-6 months	38	8.3
Wait time for first appointment (months)	7-8 months	7	1.5
Wait time for first appointment (months)	9-10 months	4	0
Wait time for first appointment (months)	10-11 months	0	0.9
Wait time for first appointment (months)	11-12 months	4	0.9
Wait time for first appointment (months)	>12 months	7	1.5
Wait times for appointments in months, mean (SD), (min., max.)		2.50 (2.49) (0.25, 12)	
Wait times for appointments in months, female, mean (SD)		2.59 (2.51)	
Wait times for appointments in months, male, mean (SD)		2.30 (2.41)	
Offices accepted all insurances (N = 453)		152 (33.6)	
Offices declined due to insurance (N = 455)		247 (54.3)	

Actual waiting times showed that 31.2% of patients secured an appointment within one month, 43% between one and three months, 55% waited more than three months, 14% waited over five months, and 3% waited nearly a year. The mean waiting time was 2.50 months (SD = 2.49), with a minimum wait of less than one month and a maximum wait exceeding 12 months.

Female patients had longer mean waiting times (2.59 months; SD = 2.51) compared with male patients (2.30 months; SD = 2.41). Although small, this difference was statistically significant (p < 0.05).

Participants were asked about the number of offices they contacted for an appointment. Of these, 21.2% contacted four to nine offices, and 4.6% contacted more than 10 offices to secure an appointment (Table 2). More than half of the participants (54.3%) attributed their limited access to appointments to a lack of dermatologists participating in their health insurance provider network. Additionally, 34.9% reported that at least half of the offices they contacted had wait times exceeding six months.

Over-the-counter medications, home remedies, and herbal medicines were also explored. The study found that 38.7% of patients reported worsening of their condition while waiting for an appointment (Table 3). Furthermore, 65.5% of participants resorted to using medications prescribed by other doctors, over-the-counter pharmacy products, home remedies, or medicinal herbs and plants to relieve their symptoms.

**Table 3:** Self-medication and impact on QOL DLQI, Dermatology Life Quality Index; HMP, herbal medicinal plant; HR, home remedies; OTC, over-the-counter; QOL, quality of life

Indicator	Value
Worsened while waiting (N = 462)	179 (38.7)
Self-treatment, any (OTC + HR + HMP) (N = 464)	285 (61.4)
HR + HMP	119 (25.6)
OTC topicals	166 (35.8)
Other prescribed topicals	79 (17.0)
Pills	42 (9.0)
HR	92 (19.8)
HMP	27 (5.8)
More than one of the above	237 (42.7)
None	198 (42.7)
Stress related to the current dermatologic condition	N = 460
None	84 (18.3)
A little	175 (38.0)
A lot + very much	201 (43.7)
Waiting for an appointment on QOL	N = 464
None	230 (49.6)
A little	141 (30.7)
A lot + very much	93 (20.0)
Waiting for an appointment for emotional health	N = 460
None + NA	175/460 (38.0)
A little	161/460 (35.0)
A lot + very much	124/460 (27.3)
DLQI score, mean (SD) (min., max.)	5.81 (6.24) (0, 27)
DLQI classifications	N = 461
Extremely large effect on QOL	19 (4.1)
Very large effect on QOL	73 (15.9)
Moderate effect on QOL	94 (20.4)
Small effect on QOL	138 (29.9)
No effect on QOL	137 (29.7)

When asked explicitly about the impact of their dermatological condition and appointment wait time on perceived stress, 38% reported experiencing stress, and 43.7% reported high levels of stress. Similarly, regarding the impact on overall emotional health, 35% of participants reported some impact, and 27% reported a significant impact. In terms of overall QOL, 31% indicated that waiting time had some impact, and 20% indicated a high impact. Scores from the DLQI instrument revealed that 40% of participants experienced moderate, large, or extremely large effects on their QOL (Table 4). 

**Table 4:** DLQI DLQI, Dermatology Life Quality Index; QOL, quality of life DLQI Spanish-PR version 1.0 (Cardiff University translation, December 11, 2013) [19].

DLQI item/band	n (%)
Q1: Symptoms: itch, pain, sting	N = 462
None	181 (39.6)
A little	148 (32.0)
A lot + very much	131 (11.9)
Q2: Embarrassed or self-conscious	N = 463
None	203 (43.8)
A little	126 (27.2)
A lot + very much	134 (28.9)
Q3: Interference with shopping, tender home/garden	N = 463
None	262 (59.6)
A little	99 (21.4)
A lot + very much	102 (22.0)
Q4: Influenced clothing selection	N = 463
None	261/463, (56.4)
A little	89/463, (19.2)
A lot + very much	102/463, (22.0)
Q5: Affected social leisure	N = 463
None	246 (53.1)
A little	104 (22.5)
A lot + very much	114 (24.4)
Q6: Unable to work/study	N = 461
Yes	38 (8.2)
No	423 (91.8)
Q7: Affects work/studying	N = 423
None	291 (68.8)
A little	79 (18.7)
A lot + very much	63 (14.9)
Q8: Affects relations with partner/friends	N = 461
None	354 (76.8)
A little	68 (14.8)
A lot + very much	39 (8.5)
Q9: Affects sexual life	N = 461
None	396 (85.9)
A little	37 (8.0)
A lot + very much	28 (6.1)
Q10: Dermatological condition treatment impact	N = 459
None + NA	373 (81.3)
A little	56 (12.2)
A lot + very much	30 (6.5)
DLQI scores	N = 461
Extremely large effect on QOL	19 (4.1)
Very large effect on QOL	73 (15.9)
Moderate effect on QOL	94 (20.4)
Small effect on QOL	138 (29.9)
No effect on QOL	137 (29.7)

Independent samples t-tests revealed two key differences in DLQI scores: women reported significantly higher scores than men (p = 0.008), and patients seeking medical dermatology services reported higher scores than those seeking cosmetic services (p < 0.001). Detailed statistics are provided in Table 5.

**Table 5:** Independent samples t-tests DLQI, Dermatology Life Quality Index

Comparison	Group 1 (mean ± SD)	Group 2 (mean ± SD)	t	df	p	Cohen’s d
DLQI: women vs. men	6.24 ± 6.46 (n = 324)	4.80 ± 5.59 (n = 137)	2.41	293.7	0.008	0.23
DLQI: medical vs. cosmetic	6.24 ± 6.37 (n = 392)	3.38 ± 4.84 (n = 68)	4.27	111.5	<0.001	0.46

Chi-square tests of independence demonstrated that the type of dermatology service was associated with worsening symptoms, DLQI severity band, perceived stress, and emotional health impact (all p ≤ 0.007). Sex showed a marginal association with waits longer than one month (p = 0.059) but was significantly related to moderate-to-severe stress (p = 0.019). Nonsignificant relationships are listed in Table 6, alongside full test statistics.

**Table 6:** Chi-square tests of independence Effect sizes are interpreted per Cohen (small ≈ 0.2; medium ≈ 0.5). DLQI, Dermatology Life Quality Index

Variables	χ²	df	p	Cramér V
Service type × worsening symptoms	7.38	1	0.007	0.13
Service type × DLQI band (moderate+ vs. < moderate)	7.89	1	0.005	0.13
Service type × stress (none/low vs. high)	21.35	1	<0.001	0.22
Sex × stress (none vs. some)	5.54	1	0.019	0.11
Sex × wait >1 month	3.55	1	0.059	0.09

However, not all relationships were significant. For instance, there was no significant relationship between patient sex and first visit (X²(1, 463) = 0.477, p = 0.490), between patient sex and type of dermatology service (X²(1, 463) = 0.735, p = 0.391), or between patient sex and history of preexisting dermatology problems (X²(1, 441) = 1.669, p = 0.196). Similarly, the relationship between patient sex and worsening of symptoms while waiting was not significant (X²(1, 462) = 0.004, p = 0.95).

## Discussion

This study is the first to explore the impact of prolonged waiting times for dermatology appointments among patients in PR, focusing on how these delays are associated with patients’ QOL, stress levels, and emotional health. The study surveyed 464 participants, revealing that 40% experienced a moderate to extreme impact on their QOL, with women and those seeking medical dermatology services reporting greater effects. Notably, 33% of patients waited more than three months for an appointment, and 40% reported worsening of their condition during the waiting period. These findings suggest that prolonged wait times are associated with adverse patient-reported outcomes, particularly among populations facing barriers to timely access to care, and may reflect broader challenges observed in healthcare systems under strain.

The study observed longer average wait times for dermatology appointments in PR (2.49 months) compared with national estimates for the US (1.13 months), highlighting potential disparities in access to dermatological care. Factors associated with reduced access to dermatology services in PR appear similar to those reported in mainland US studies, including sex, race/ethnicity, income, educational level, insurance status, rurality, and self-reported health condition [20,21]. In this study, health insurance emerged as an important factor influencing access to dermatological appointments. The proportion of participants with private health insurance (57%) was higher than that of the general PR population, while those with government-sponsored insurance (18%) or no insurance (2%) were underrepresented [22], suggesting that individuals with limited insurance coverage may face greater barriers to obtaining care.

Workforce availability likely contributes to access challenges in PR; however, precise data regarding the number and geographic distribution of practicing dermatologists on the island remain limited. An estimated ratio of 1.85 general dermatologists per 100,000 inhabitants represents approximately a 50% deficit relative to the recommended US threshold of 3.66 per 100,000 [23]. Nationally, dermatology has experienced persistent workforce shortages and geographic maldistribution favoring urban areas since the late 1990s, trends that may also be relevant in PR. Limited financial capacity to support graduate medical education and specialty training on the island may further constrain workforce expansion. Although these factors plausibly contribute to access disparities, this study was not designed to directly assess causality between workforce distribution and appointment wait times.

Participants residing outside metropolitan areas constituted the majority of the study sample; however, it was not possible to reliably estimate the number of participants living in rural areas based on the available data. In PR, rural classification is largely determined by population density and may not accurately reflect true geographic isolation or the availability of healthcare resources. As a result, comparative analyses of wait times across rural and urban regions could not be performed. This limitation highlights the need for future studies incorporating more granular geographic and workforce data to better characterize regional access disparities. National data indicate that dermatologist density decreases substantially outside metropolitan areas, with many counties lacking dermatology services altogether [24,25], a pattern that may be relevant to PR but cannot be directly inferred from the present study.

A substantial proportion of participants reported using over-the-counter medications and home remedies while awaiting care, which may serve as an indirect indicator of delayed access to dermatological services. Prolonged waiting times were associated with worse patient-reported QOL, increased stress, and emotional burden. Women, in particular, reported higher stress levels and greater QOL impact, although this finding should be interpreted in light of the higher proportion of female participants in the study sample. Significant associations between medical dermatology visits, worsening symptoms, stress, and QOL impact were observed, consistent with existing literature emphasizing the importance of timely access to dermatologic care in managing chronic conditions [23]. While these findings suggest that delays in care may contribute to adverse patient behaviors such as self-medication, causal inferences cannot be drawn from this cross-sectional design.

## Conclusions

This study highlights the significant challenges posed by a suboptimal dermatology workforce and socioeconomic factors, particularly amid rising demand for services, and raises concerns about how health insurance plans may exacerbate these issues. It validates patient complaints regarding long wait times and unequal access to dermatology appointments, representing the first exploration of the impact of appointment wait times on patients in PR. The findings emphasize the need for ongoing monitoring and strategic interventions to ensure a sufficient supply of dermatology professionals and to develop public policies that protect patients’ health. Addressing these disparities requires a multifaceted approach, including increasing the number of dermatologists in underserved areas, investing in healthcare infrastructure, and assessing the impact of these measures on health outcomes and costs. Collaborative efforts among government agencies, healthcare organizations, academia, and community stakeholders are essential for implementing sustainable solutions that ensure equitable access to dermatological care for all residents of PR. Integrating healthcare data from US territories, such as PR, into national discussions is critical for reducing disparities and improving healthcare quality across the US, thereby advancing broader goals of public health equity.

## Appendices

Dermatology Appointments Waiting Time Questionnaire - English

Dear participant,

This study consists of a survey for patients about their initial visit to a dermatologist. The survey aims to estimate the average time it takes to get an initial evaluation appointment with a dermatologist in Puerto Rico and the factors that affect this process. The questionnaire will explore physical and emotional aspects related to the waiting time for a dermatology appointment in PR. The study aims to provide valuable information on the accessibility of healthcare and make recommendations to healthcare providers in Puerto Rico, which may lead to improving access and availability to dermatological care.

Please complete the survey below. Thank you!

Dermatology Research Team

1) Consent/assent to participate in the research study

Yes

No

By signing this document, you agree to participate in this study. Please make sure you understand the details of the study before signing. The office staff should have provided you with a written consent form explaining the purpose of the study. Please keep a copy of this document for your records. If you have any questions about the study after signing this document, you can contact the research team using the information provided in the papers you were given. By this means, I certify that I understand the details of the study; my questions so far have been answered and clarified, and I agree to participate in it.

2) This questionnaire is being answered by an adult patient (>18 years old)

Minor patient (< 18 years old)

Family member or caregiver

Office staff

Study personnel

3) Is this your first visit to this dermatology office?

Yes

No

4) Age of the patient:

5) Sex at birth:

Female

Male

Intersex

Prefer not to answer

6) Gender identity:

Male

Female

Non-binary

Transgender

Prefer not to answer

7) Are you Hispanic/Latino?

Yes

No

8) Race:

White (Caucasian)

Black (African American)

Native American or Alaska Native (American Indian)

Asian

Pacific Islander or Hawaiian

Mixed race

Prefer not to answer

9) Zip code:

10) Town/city where you live:

Adjuntas

Aguada

Aguadilla

Aguas Buenas

Aibonito

Añasco

Arecibo

Arroyo

Barceloneta

Barranquitas

Bayamón

Cabo Rojo

Caguas

Camuy

Canóvanas

Carolina

Cataño

Cayey

Ceiba

Ciales

Cidra

Coamo

Comerío

Corozal

Culebra

Dorado

Fajardo

Florida

Guánica

Guayama

Guayanilla

Guaynabo

Gurabo

Hatillo

Hormigueros

Humacao

Isabela

Jayuya

Juana Díaz

Juncos

Lajas

Lares

Las Marías

Las Piedras

Loíza

Luquillo

Manatí

Maricao

Maunabo

Mayagüez

Moca

Morovis

Naguabo

Naranjito

Orocovis

Patillas

Peñuelas

Ponce

Quebradillas

Rincón

Río Grande

Sabana Grande

Salinas

San Germán

San Juan

San Lorenzo

San Sebastián

Santa Isabel

Toa Alta

Toa Baja

Trujillo Alto

Utuado

Vega Alta

Vega Baja

Vieques

Villalba

Yabucoa

Yauco

11) Which of the following health insurance plans do you I don't have health insurance have? Please select all that apply:

Medicare

MMM

Reforma - Vital

Private Insurance

12) Estimated date when you made this dermatology appointment (month-day-year)

13) How many dermatologist offices did you call before getting an appointment?

1 to 3

4 to 6

7 to 9

10 or more

I only called this one.

14) In how many of the offices did the appointments exceed only one, with a six-month wait for new patients?

Some of them (2 or 3 of them)

Quite a few (half of the ones I called)

Almost all or the majority of them

None exceeded six months waiting period

15) Did any office not accept your health insurance plan?

In only one

In some (2 or 3 of them)

Quite a few (half of the ones I called)

Almost all or the majority

All the ones I called accepted my health insurance plan

16) How much time has passed from the day you made this appointment until today?

1-6 days

7-14 days

15-21 days

22-28 days

29-31 days

2-3 months

4-5 months

6-7 months

8-9 months

10-11 months

12 or more months

17) Did any office refuse to schedule an appointment because they didn't have availability for new patients?

Yes

No

18) Is this the first time you have a dermatological condition?

Yes

No

19) What prompted you to schedule an appointment with a dermatologist?

A medical - dermatological issue

An aesthetic or cosmetic concern

Both a dermatological issue and a cosmetic concern

20) Do you feel that the concern for which you requested the appointment worsened while waiting for the day of this medical appointment?

Yes

No

21) Did you use any over-the-counter medication or home remedies while waiting for this appointment?

Yes

No

22) If you answered "yes" to the previous question, please specify what you used. Check all that apply.

Non-prescription creams

Prescription creams from other doctors

Pills

Home or natural remedies

Medicinal plants or herbal medicine

I did not use over-the-counter medications or remedies for my dermatologic symptoms.

23) Over the last week, how itchy, sore, painful, or stinging has your skin been?

Very much

A lot

A little

Not at all

24) Over the last week, how embarrassed or self-conscious have you been because of your skin?

Very much

A lot

A little

Not at all

25? Over the last week, how much has your skin interfered with you going shopping or looking after your home or yard?

Very much

A lot

A little

Not at all

26) Over the last week, how much has your skin influenced the clothes you wear?

Very much

A lot

A little

Not at all

27) Over the last week, how much has your skin affected any social or leisure activities?

Very much

A lot

A little

Not at all

28) Over the last week, how much has your skin made it difficult for you to do any sport?

Very much

A lot

A little

Not at all

29? Over the last week, has your skin prevented you from working or studying?

Yes

No

30) If "No", over the last week, how much has your skin been a problem at work or studying?

A lot

A little

Not at all

31) Over the last week, how much has your skin created problems with your partner or any of your close friends or relatives?

Very much

A lot

A little

Not at all

32) Over the last week, how much has your skin caused any sexual difficulties?

Very much

A lot

A little

Not at all

33) Over the last week, how much of a problem has the treatment for your skin been, for example, by making your home messy, or by taking up time?

Very much

A lot

A little

Not at all

34) How much has the dermatological problem affected your quality of life during the waiting time for this appointment?

Very much

A lot

A little

Not at all

35) How much stress does having this dermatological problem cause you?

Very much

A lot

A little

Not at all

36) What impact has the wait to address your skin health condition had on your emotional well-being?

Very much

A lot

A little

Not at all

37) How would you feel if you had to live the rest of your life with the symptoms for which you visited the dermatologist today?

Very satisfied

Satisfied

Slightly satisfied

Equally satisfied as dissatisfied

Dissatisfied

Very dissatisfied

Terrible

Doesn't apply

## References

[1] Roman J (2015). The Puerto Rico healthcare crisis. Ann Am Thorac Soc.

[2] Allen G (2025). SOS: Puerto Rico is losing doctors, leaving patients stranded. https://www.npr.org/sections/health-shots/2016/03/12/469974138/sos-puerto-rico-is-losing-doctors-leaving-patients-stranded.

[3] McIntyre D, Chow CK (2020). Waiting time as an indicator for health services under strain: a narrative review. Inquiry.

[4] (2025). Puerto Rico Physician Workforce Profile.

[5] Garcovich S, Colloca G, Sollena P (2017). Skin cancer epidemics in the elderly as an emerging issue in geriatric oncology. Aging Dis.

[6] Matos-Moreno A, Verdery AM, Mendes de Leon CF, De Jesús-Monge VM, Santos-Lozada AR (2022). Aging and the left behind: Puerto Rico and its unconventional rapid aging. Gerontologist.

[7] Gronbeck C, Kodumudi V, Brodell RT, Grant-Kels JM, Mostow EN, Feng H (2023). Dermatology workforce in the United States — part I: overview, transformations, and implications. J Am Acad Dermatol.

[8] Huq F, Nakamura M, Black K, Chubb H, Helfrich Y (2020). Association of dermatology wait times with insurance coverage in Michigan. Am J Manag Care.

[9] Creadore A, Desai S, Li SJ (2021). Insurance acceptance, appointment wait time, and dermatologist access across practice types in the US. JAMA Dermatol.

[10] Glazer AM, Rigel DS (2017). Analysis of trends in geographic distribution of US dermatology workforce density. JAMA Dermatol.

[11] Xiang L, Lipner SR (2020). Analysis of wait times for online dermatology appointments in most and least dermatologist-dense cities. J Drugs Dermatol.

[12] (2025). Survey of Physician Appointment Wait Times and Medicare and Medicaid Acceptance Rates.

[13] (2025). Patients Are Waiting: America’s Dermatology Wait Times Crisis.

[14] Mazmudar RS, Gupta N, Desai BJ, Bordeaux JS, Scott JF (2020). Dermatologist appointment access and waiting times: a comparative study of insurance types. J Am Acad Dermatol.

[15] Nielsen ML, Nymand LK, Thomsen SF, Thyssen JP, Egeberg A (2022). Delay in diagnosis and treatment of patients with psoriasis: a population‐based cross‐sectional study. Br J Dermatol.

[16] Kodumudi V, Gronbeck C, Brodell RT, Grant-Kels JM, Mostow EN, Feng H (2023). Dermatology workforce in the United States - part II: patient outcomes, challenges, and potential solutions. J Am Acad Dermatol.

[17] Suneja T, Smith ED, Chen GJ, Zipperstein KJ, Fleischer AB, Feldman SR (2001). Waiting times to see a dermatologist are perceived as too long by dermatologists: implications for the dermatology workforce. Arch Dermatol.

[18] Finlay AY, Khan GK (1994). Dermatology Life Quality Index (DLQI)—a simple practical measure for routine clinical use. Clin Exp Dermatol.

[19] (2025). Dermatology Life Quality Index (DLQI) v1.0 Translation Paper (Level 2-LVT): Spanish (Puerto Rico). https://www.cardiff.ac.uk/medicine/resources/dermatology-questionnaires.

[20] Tripathi R, Knusel KD, Ezaldein HH, Scott JF, Bordeaux JS (2018). Association of demographic and socioeconomic characteristics with differences in use of outpatient dermatology services in the United States. JAMA Dermatol.

[21] Alghothani L, Jacks SK, Vander Horst A, Zirwas MJ (2012). Disparities in access to dermatologic care according to insurance type. Arch Dermatol.

[22] Shah M, Burshtein J, Zakria D, Rigel D (2024). Analysis of trends in US dermatologist density and geographic distribution. J Am Acad Dermatol.

[23] Porter ML, Kimball AB (2018). Predictions, surprises, and the future of the dermatology workforce. JAMA Dermatol.

[24] (2025). Puerto Rico population (live). https://www.worldometers.info/world-population/puerto-rico-population/.

[25] Vaidya T, Zubritsky L, Alikhan A, Housholder A (2018). Socioeconomic and geographic barriers to dermatology care in urban and rural US populations. J Am Acad Dermatol.

